# REMEDIATION: The Gene behind Arsenic Hyperaccumulation

**DOI:** 10.1289/ehp.118-a337

**Published:** 2010-08

**Authors:** Carol Potera

**Affiliations:** **Carol Potera**, based in Montana, has written for *EHP* since 1996. She also writes for *Microbe*, *Genetic Engineering News*, and the *American Journal of Nursing*

*Pteris vittata* (brake fern) has been shown to accumulate large amounts of arsenic taken up from soil,^1^ in one study removing more than a quarter of the soil arsenic within 20 weeks.^2^ Now researchers have isolated the gene responsible for this feat: *ACR3*, which encodes a protein that pumps the metal into the vacuoles of plant cells.^3^ “Plants sequester toxicants in these vacuoles—we call them the plant’s trash can,” says principal investigator Jo Ann Banks, a professor of botany at Purdue University.

*ACR3* is an arsenite efflux transporter gene found only in gymnosperms (nonflowering plants).^3^ Banks and horticulturist David Salt, also of Purdue University, identified *ACR3* in *P. vittata* by using a mutant yeast strain that lacks *ACR3* and dies when exposed to arsenic. The team inserted thousands of genes from *P. vittata* and found the one that corrected the deficiency, allowing the mutant to tolerate arsenic. They also showed that arsenic exposure stimulated *ACR3* activity. Fern gametophytes grown in an arsenic-laced medium produced 35 times more *ACR3* transcripts than those grown without arsenic. Moreover, ferns grown hydroponically in arsenic medium confirmed that *ACR3* activity was also highly induced in the roots.

As for what happens when the arsenic-laden plants die, Banks says, “The plants are ashed or composted to reduce biomass. There are a few labs researching how to convert the leftover arsenic into nontoxic organic arsenic compounds.”

Ferns are not the only plants that sequester arsenic. Crops such as rice have been shown to accumulate levels of arsenic high enough to threaten human health,^4^ making it important to learn how plants transport, store, and tolerate arsenic. Such information could lead to ways to manipulate rice plants to restrict arsenic to the roots and prevent contamination of edible grains. “Or we may even devise a way to keep rice plants from taking up arsenic at all,” says Banks.

“If this gene can be cloned into problematic crops such as rice, arsenic burdens in edible parts may be greatly reduced,” agrees Andrew Meharg, chair of biogeochemistry at the University of Aberdeen, United Kingdom. He adds that the new study “is a major advance in our understanding of how plants that concentrate high levels of arsenic are able to tolerate the toxic element.”

Landscapers currently plant *P. vittata* to clean up soils contaminated with arsenic from pesticides and pressure-treated lumber.^5^ However, the fern naturally grows only in warm climates such as Florida. Perhaps cold-tolerant plants could be programmed with *ACR3* to hyperaccumulate arsenic, too. Joseph Graziano, a professor of environmental health at Columbia University in New York City, notes, “It seems possible that the discovery of this gene could lead to the creation of genetically modified plants or trees with the ability to remove significant amounts of arsenic from contaminated soils.”

## Figures and Tables

**Figure:**
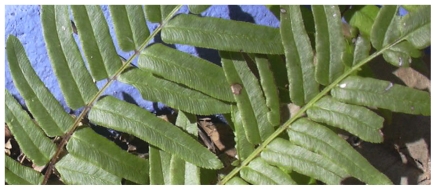
Pteris vittata
